# The effect of minimally invasive suturectomy with postoperative cranial remolding orthotic treatment for an infant with bilateral coronal craniosynostosis

**DOI:** 10.1002/ccr3.7692

**Published:** 2023-07-11

**Authors:** Zahra Taheri, Taher Babaee, Hassan Reza Mohammadi, Behnam Hajiaghaei, Alireza Khani

**Affiliations:** ^1^ Department of Orthotics and Prosthetics, Rehabilitation Research Center, School of Rehabilitation Sciences Iran University of Medical sciences Tehran Iran; ^2^ Department of Neurosurgery Shaheed Beheshti University of Medical Sciences Tehran Iran; ^3^ Department of Orthotics and Prosthetics University of Social Welfare and Rehabilitation Tehran Iran

**Keywords:** compliance, craniosynostosis, orthosis, remolding, suturectomy

## Abstract

Minimally invasive suturectomy has been reported to significantly decrease the economic cost of treating infants with craniosynostosis. Nonetheless, treatment should be accompanied by a cranial remolding orthosis to maintain the constant correction and reshaping of the skull throughout the infant's development.

## 1 INTRODUCTION

The early‐onset closure of one or more of the cranial sutures (CS) referred to as craniosynostosis may be present due to unknown causes, syndromic reasons, or familial conditions.^1^ Depending on which CS being fused, the morphology of the cranium differs. The prevalence of craniosynostosis is 5.9 per 10,000 live births.^2^

The majority of craniosynostosis cases are single‐CS non‐syndromic type, but multiple‐CS synostosis has also been observed.^3^ The fusion of bilateral coronal CSs is the most common type of multisutural craniosynostosis that causes brachycephaly and is often associated with midface hypoplasia.^4^ The term “brachycephaly” is derived from the Greek words “brakhu” (short) and “cephalos” (head), which translates to “short head,” referring to the premature obliteration of the bilateral coronal CSs.^5^ Bilateral flattening of the occiput in infants with this condition results in a smaller anteroposterior cranial dimension and a larger mediolateral dimension.^6^ This condition also leads to a vertically tall (towered‐shaped) skull.^7^ These cases necessitate a more complex treatment; for cases with a late diagnosis, more than one surgery is required to restore the normal shape of the head.^5^

Endoscopic‐assisted craniectomy is a minimally invasive procedure that allows brain growth by removing an abnormally fused CS.^8^ Age has been reported to be a predictor of treatment success with patients under the age of 5 or 6 months demonstrating an optimal outcome.^4^ The existing body of research suggests that minimally invasive suturectomy accompanied by a postoperative cranial remolding orthosis (CRO) has proven safe and successful in treating patients with craniosynostosis, resulting in positive outcomes and improvements in cranial deformity and symmetr.^9^, ^10^, ^11^ Less blood transfusions, reduced surgery time, and lesser hospitalization days are advantages of minimally invasive suturectomy.^12^ This procedure creates a wide sutural excision in conjunction with lateral osteotomies so that future growth of the cranium occurs in all the three dimensions. However, the optimal three‐dimensional growth of the patient's cranium can be achieved by using a properly fitted CRO on the patient's head.^13^ Data from several studies suggest that improvement in proportion and symmetry of the cranium is inversely related to the age at which the procedure is performed.^11^, ^14^

This report represents the efficacy of endoscopic assisted suturectomy followed by postoperative CRO treatment on cranial symmetry of an infant with bilateral coronal craniosynostosis with an extremely severe skull deformity.

## 2 CASE PRESENTATION

**FIGURE 1:**
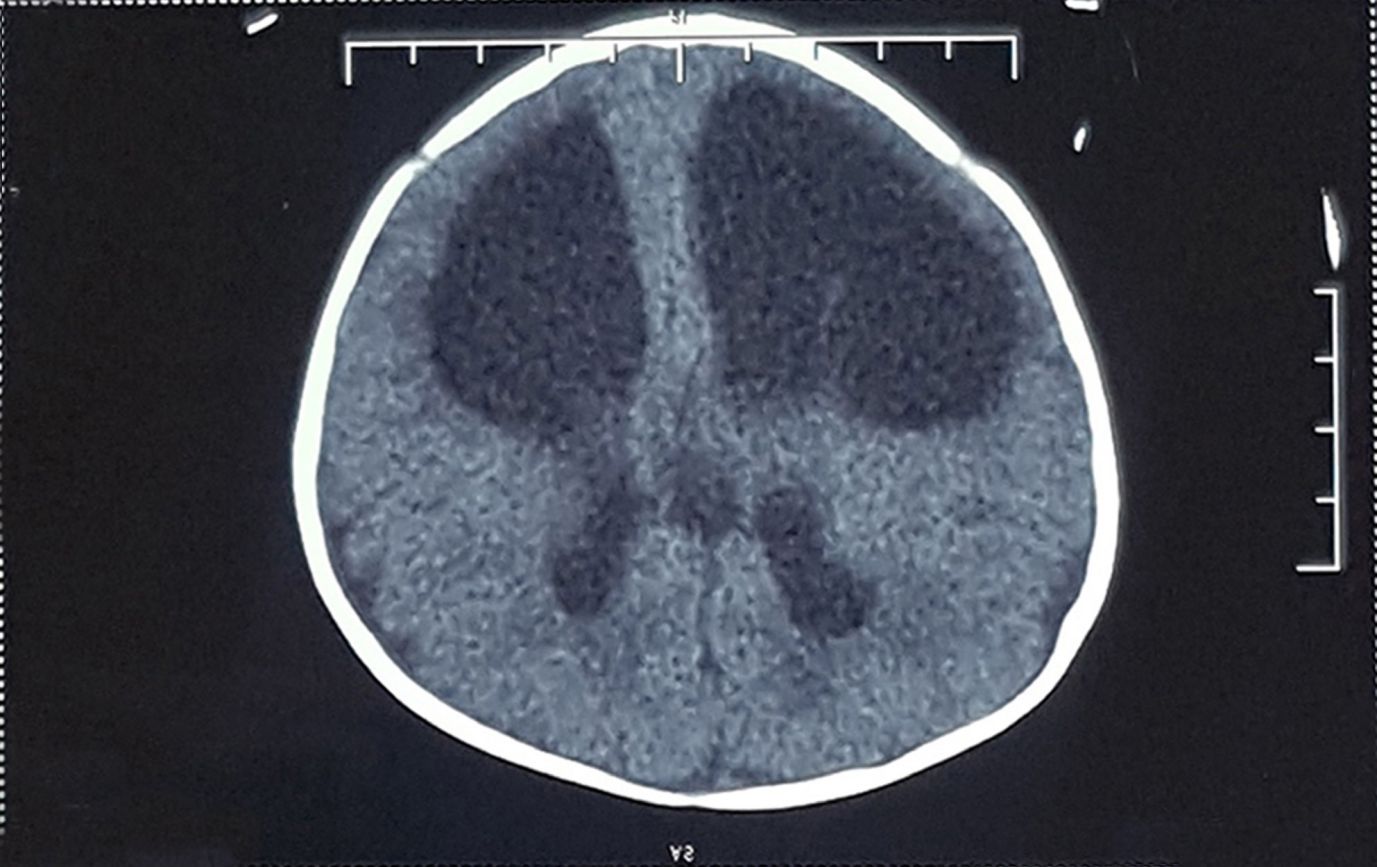
The computer tomography image of the patient skull.

A 55‐day‐old boy infant with extremely severe brachycephaly was referred to our clinic. The patient was delivered at 38 weeks' gestation by a 29‐year‐old mother through a cesarean section. The patient had a routine intrauterine course without any complication. He had a 10‐days history of being admitted to the neonatal intensive care unit because of abnormal head shape. CT images revealed immature fusion of both coronal CS and syndromic synostosis in the infant (Figure 1). Before commencing treatment, the patient exhibited poor motility and a history of three convulsions due to elevated intracranial pressure. Consequently, a craniectomy with postoperative CRO therapy was recommended. At the start of the procedure, the cranial index (CI) and cranial vault asymmetry index (CVAI) rates were 130% and 5.2%, respectively.

### 2.1 Surgical intervention and CRO fitting process

After general anesthesia, the patient was positioned in supine, with head in neutral position on a horseshoe headrest. Incisions were made bilaterally, 2 cm above the fused CSs, from the anterior fontanel to the level of the lateral canthus. Subsequently, electrocautery was used to create an incision plane between the galea and pericranium, revealing the entire fused coronal sutures under endoscopic vision with a rigid lens. A subcutaneous burr hole was then created, and a 1‐cm‐wide strip craniectomy was performed with a rongeur along the bridged CS. Subsequent epidural incisions made inferiorly under endoscopic magnification exposed the pterion and lesser wing of the sphenoid. Suturectomy was continued down to unlock pterional area, allowing the eye to move forward and down.^10^, ^11^ The incisions were then fastened using standard method with absorbable stitches.

Ten days after the surgery and the swelling subsiding, a bivalve CRO was fitted to the patient's head. A noncontact blue‐light 3D optical scanner (Vorum, SPECTRA 3D, Vancouver, Canada) with a precision of 0.1 mm was utilized to obtain a representative model of the infant's head. The resulting cranial image was then imported into the Vorum orthotics and prosthetics design software to generate a positive model of the infant's cranium. Using this software, we precisely applied modifications to achieve a more symmetrical head shape and to increase the original cranium's proportion. Flattening regions, such as the frontal and occipital regions, were enlarged to accommodate skull growth in the anterior–posterior direction, while the lateral bossing in the temporal regions and the towered region on the top of the head were preserved to prevent further growth. Afterward, the positive mold was developed using a computer‐aided manufacturing lathe. First, a 10 mm layer of closed‐cell polyethylene medical foam was applied to the positive model to construct the CRO. Subsequently, a 5‐mm polypropylene plastic was draped over the foam. When the plastic and foam were separated from the mold, the CRO was cut according to preparatory trim lines and the borders were smoothly polished to avoid potential skin scratch. After wearing the CRO for the first time, we finalized its fitting on the patient's head.

The CI and CVAI are the most commonly used anthropometric measures to determine improvement of cranial deformity and symmetry of the cranium. CI is derived from the cranial width to cranial length ratio multiplied by 100, and its normal range varies from 82% to 83%.^15^ CVAI is the difference between the long and short cranial diagonal diameter divided by the long diagonal diameter multiplied by 100, and a CVAI of less than 3% is deemed normal.^1^

**FIGURE 2:**
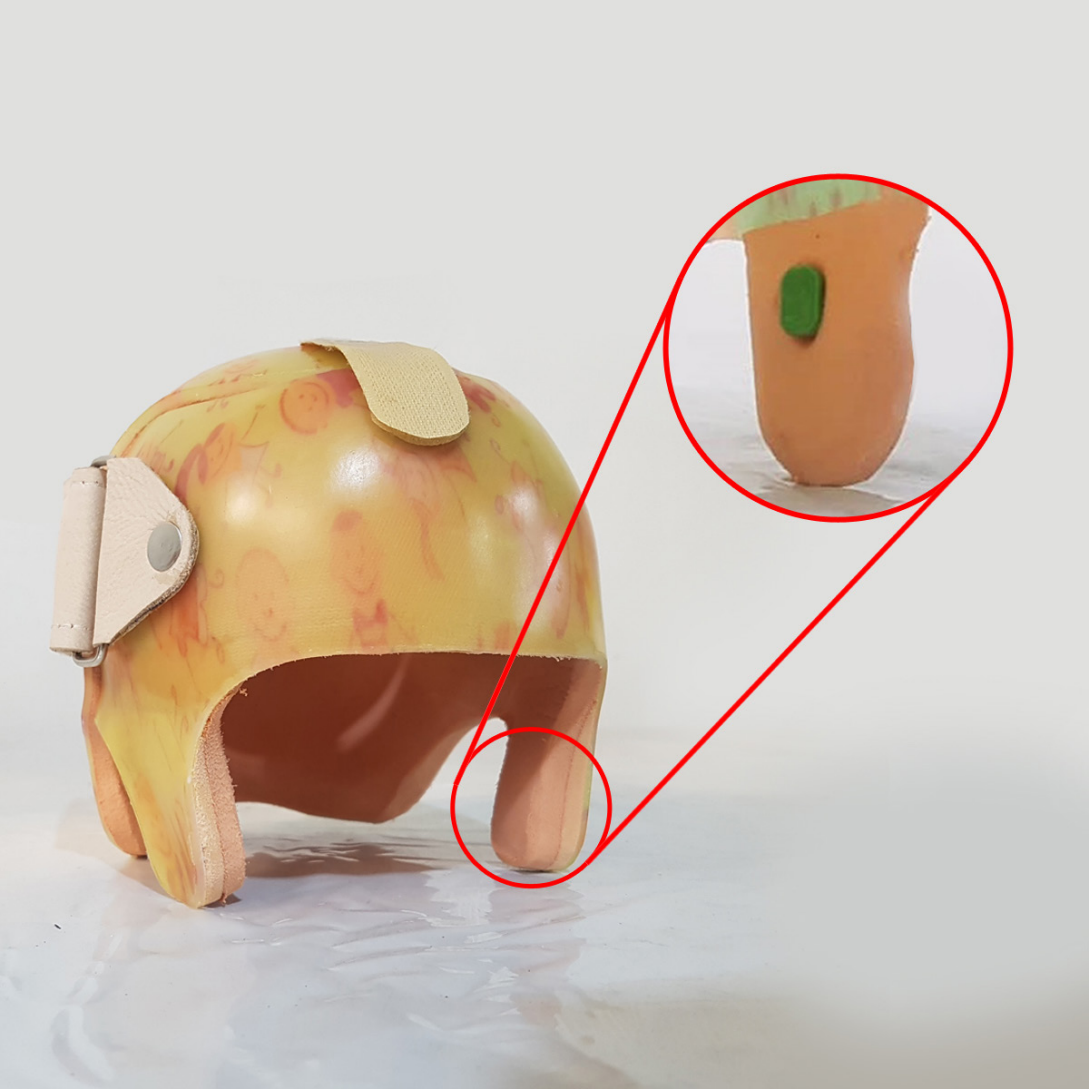
The cranial remolding orthosis with an embedded sensor data logger.

After fitting the CRO, we measured and recorded the anthropometric values of the patient's skull with a measuring tape and a caliper at each visit. The parents were instructed to wear the CRO for a minimum of 23 h per day. The CRO wearing time was objectively and subjectively measured. To evaluate objective usage, a miniature temperature logger (Orthotimer®, Rollerwerk medical engineering, Balingen, Germany) was embedded into the temporal extension part of the CRO to track the actual CRO wearing time (Figure 2). To evaluate subjective usage, the parents were requested to record the daily CRO wearing time in a logbook. The parents were instructed to record the daily CRO wearing time in a logbook to assess subjective usage. In addition, parents were requested to complete a visual analog scale‐based questionnaire regarding any CRO‐related issues.^16^ A written informed consent was obtained from the patient's parents before taking part in this study and confirmed the figures for publication.

**FIGURE 3:**
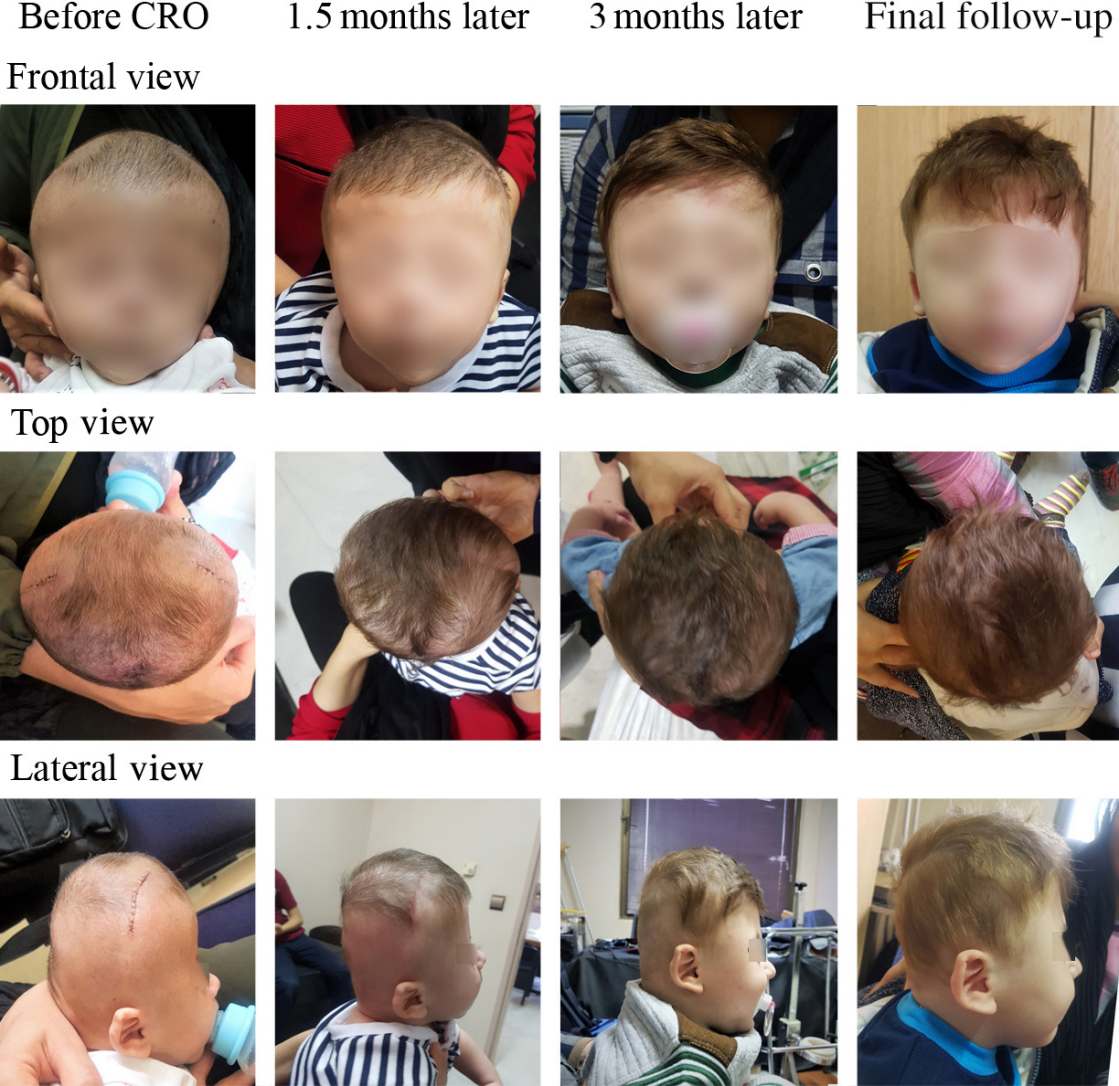
Improvements in the patient's cranium in the course of cranial remolding orthosis treatment.

**FIGURE 4:**
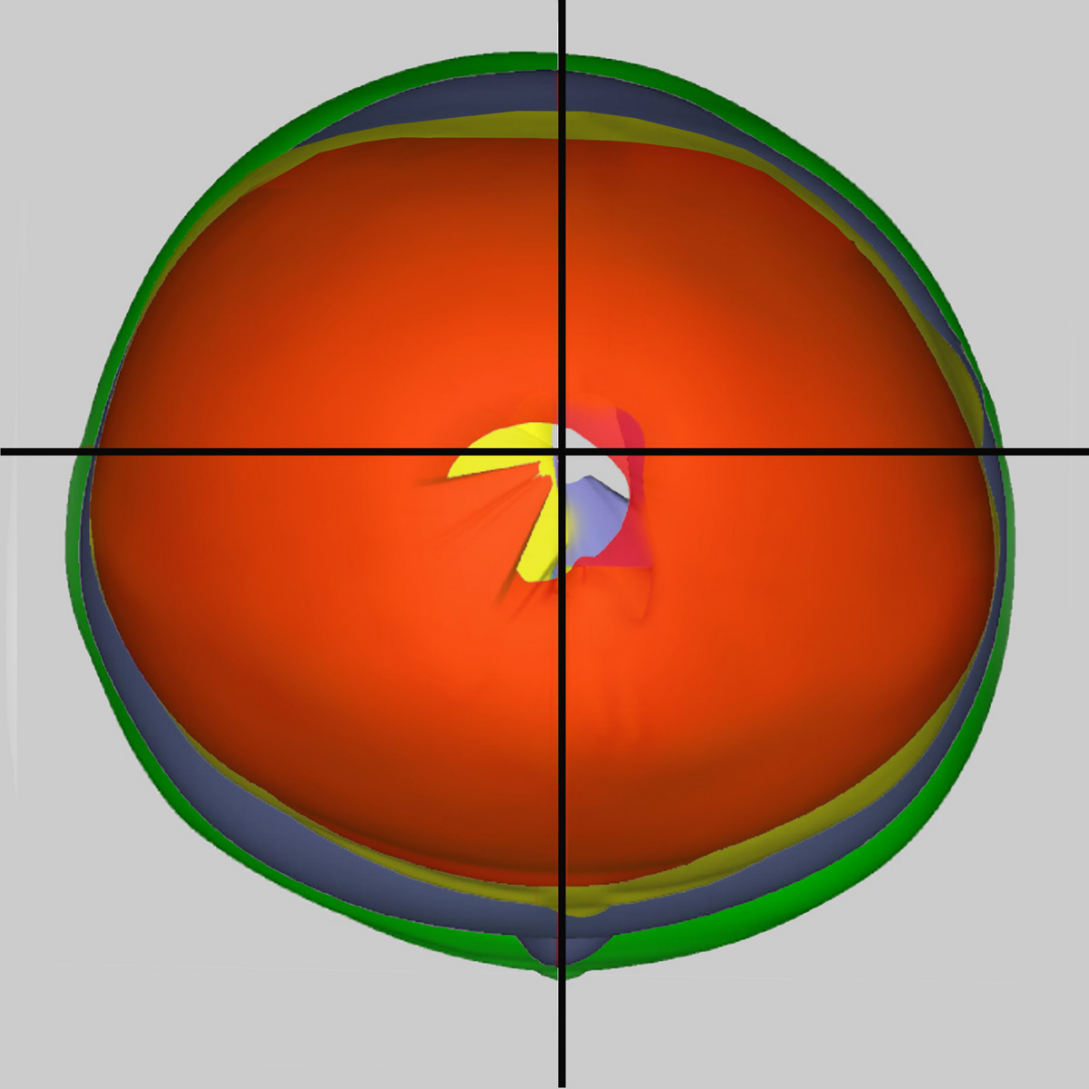
The 3D scan of the patient's cranium from the initiation to the end of treatment (top view).

**FIGURE 5:**
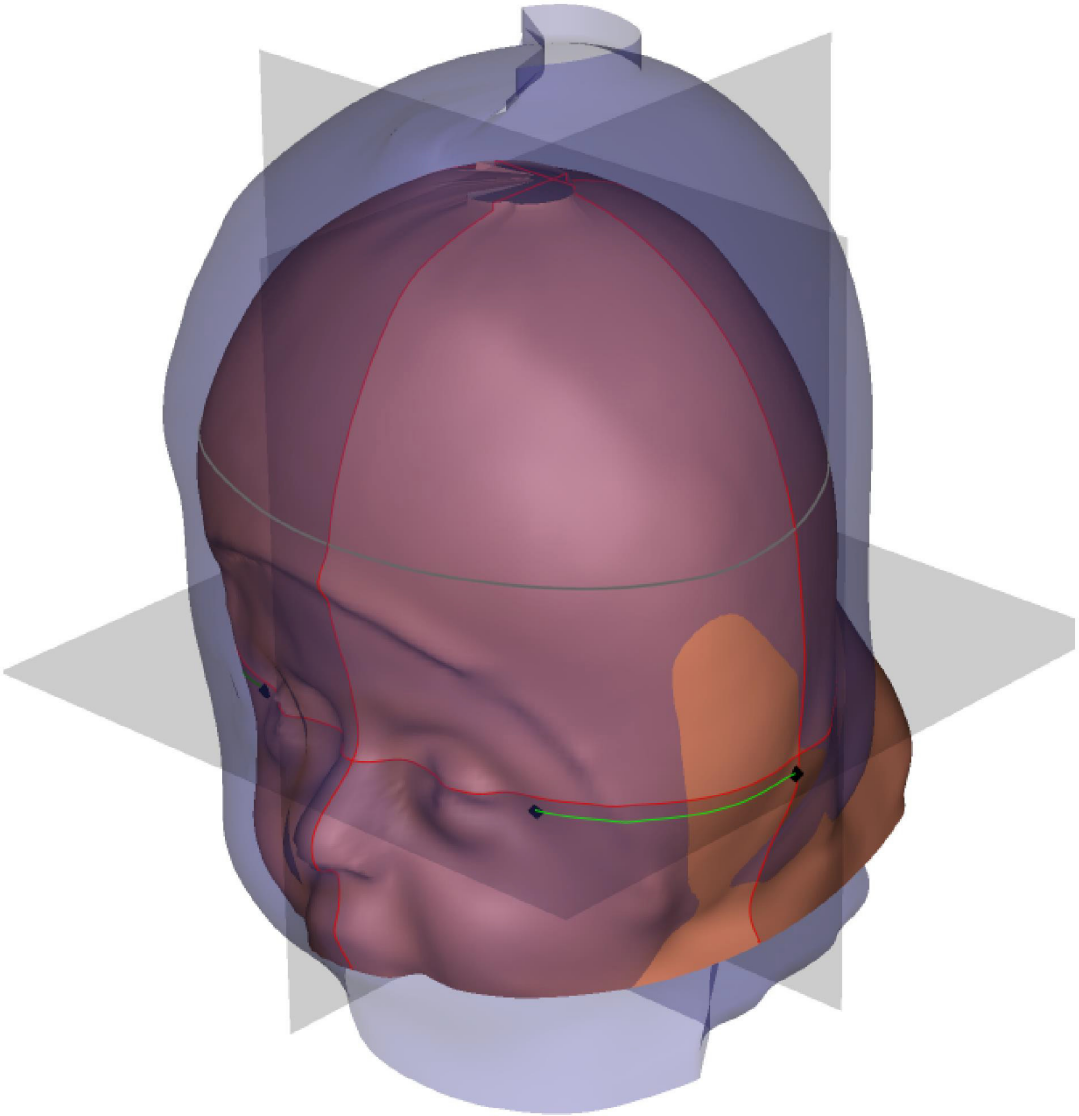
Semitransparent three‐dimensional model of the patient's cranium at the initiation and end of the CRO treatment.

The surgery lasted 90 min and blood loss was 35 mL. The infant's hospitalization lasted for 2 days. There was no major postoperative complication to require readmission. The infant was treated with CRO for 8 months. However, the anthropometric follow‐up was performed till the infant was 13 months old. Four CROs were made in total during the treatment process. After completing the CRO treatment, the CI and CVAI reached 98% and 0.7%, respectively. Figures 3, 4 and 5 reveals improvements in the patient's cranium during CRO treatment. The average daily use of a CRO was 19.31 h, according to data derived from subjective evaluations of wearing time. However, the data logger sensor showed that the average daily wearing time was only 16.5 h, less than the time the parents had reported.

## 3 DISCUSSION

Most research on postoperative CROs includes single CS synostosis.^10^, ^11^, ^13^, ^14^ The efficacy of postoperative CROs in treating multiple CS synostosis is debatable, and insufficient data support their use. Littlefield reported that the probability of re‐surgery for patients with craniosynostosis increases if postoperative CROs are not administered.^17^ In the current study, despite a remarkable decrease in CI value from 130% to 98%, based on the reported normal CI value of 82% to 83% for age‐matched children,^15^ this remains outside the normal range. According to the head proportional measurements, specifically the CI, and personal judgment from a cosmetic point of view, the skull's symmetry improvement is optimum in most cases with sagittal synostosis and relatively good in those with bilateral coronal synostosis.^10^, ^11^ In their study, Rottgers et al.^4^ found a significant improvement in the mean CI during the treatment course from 0.94 to 0.84 in a sample of patients with bilateral coronal craniosynostosis. Therefore, the postoperative CRO treatment is an effective strategy for reshaping the skull in infants with bilateral coronal craniosynostosis.

The use of CRO may have some complications such as pressure sores, ethanol erythema, loose fitting, and difficulties in terms of donning and doffing. According to parental reports, displacement of the CRO on infant's head, and perspiration were the chief complaint in using the orthosis.

CRO compliance is one of the important factors for obtaining the desired outcome of treatment in infants with asymmetrical craniums.^18^ According to the objective assessment of CRO usage, we observed that overall CRO‐wearing time was 16.5 h which was less than that of the recommended time. However, the average of subjectively reported wearing time was 19.31 h per day. Evidently, the parents overestimated the duration of CRO use. This finding is consistent with data reported in other studies on objective monitoring of spinal braces wear in adolescents with idiopathic scoliosis.^19^ Further research is required to investigate the relationship between CRO compliance and cranial correction or remodeling rate in infants with craniosynostosis. The key clinical message of this report is that minimally invasive craniectomy accompanied by a properly fitted CRO can be effective in reshaping a severely asymmetrical skull due to bilateral coronal craniosynostosis. This method eliminates the need for secondary surgery and its potential complications; therefore, it should be considered as treatment of choice.

## 4 CONCLUSION

Minimally invasive craniectomy with a minimum of 16.5 h per day postoperative CRO treatment is an effective approach for managing infants with an extremely severe deformity induced by bilateral coronal craniosynostosis.

## AUTHOR CONTRIBUTIONS

**Zahra Taheri:** Conceptualization; data curation; methodology; software; writing – original draft; writing – review and editing. **Taher Babaee:** Conceptualization; data curation; investigation; methodology; project administration; resources; software; supervision; writing – review and editing. **Hassan Reza Mohammadi:** Conceptualization; data curation; methodology; resources; validation; writing – review and editing. **Behnam Hajiaghaei:** Conceptualization; data curation; methodology; resources; software; writing – review and editing. **Alireza Khani:** Conceptualization; methodology; writing – original draft; writing – review and editing.

## FUNDING INFORMATION

The authors received no financial support for the work described in this article. The study was approved by the ethics Committee of Iran University of Medical Sciences (No. 1398.178).

## CONFLICT OF INTEREST STATEMENT

The authors have no conflict of interest to declare.

## ETHICAL STATEMENT

This study was performed in accordance with the Helsinki declaration. Data published anonymously.

## CONSENT

Written informed consent was obtained from the patient's parents to publish this report in accordance with the journal's patient consent policy.

## Data Availability

Data sharing is not applicable to this article, as no datasets were generated or analyzed during the current study.
